# Acute angle closure glaucoma from spontaneous massive subretinal hemorrhage

**DOI:** 10.3205/oc000104

**Published:** 2019-04-04

**Authors:** George Michael N. Sosuan, Rolando Enrique D. Domingo

**Affiliations:** 1 Department of Ophthalmology and Visual Sciences, Sentro Oftalmologico Jose Rizal, University of the Philippines, Manila – Philippine General Hospital, Manila, Phillipines

**Keywords:** subretinal hemorrhage, angle closure glaucoma, retinal detachment

## Abstract

**Purpose:** To report a case of acute angle closure glaucoma from spontaneous massive hemorrhagic retinal detachment from hypertension and diabetes mellitus.

**Methods:** A 52-year-old woman with controlled systemic hypertension and newly diagnosed diabetes mellitus presented with sudden onset painful loss of vision of the right eye. Examination of the right eye showed no light perception, an intraocular pressure of 60 with shallow anterior chamber, microystic corneal edema, closed angles on gonioscopy, and massive subretinal hemorrhage on indirect ophthalmoscopy. The left eye was essentially normal except for arterio-venous crossing changes. Ocular ultrasound revealed massive subretinal hemorrhage with possible intraocular mass. Enucleation of the painful blind eye was done for histologic diagnosis.

**Results:** The ocular pathology revealed complete angle closure with total retinal detachment from massive subretinal hemorrhage with no mass or tumor seen. Metastatic work-up included liver enzymes, mammography, transvaginal ultrasound, chest radiography, and cranial and abdominal computerized tomography which were all normal.

**Conclusion:** Hypertension and diabetes mellitus may cause spontaneous massive subretinal hemorrhagic retinal detachment resulting in secondary angle closure glaucoma. Enucleation is a therapeutic option if a suspicion of an intraocular tumor is present.

## Introduction

An accumulation of blood between the neurosensory retina and the retinal pigment epithelium causes subretinal hemorrhage. Blood may come from the retinal or choroidal circulation. The causes of subretinal hemorrhage are as follows:

- choroidal neovascularization associated with age-related macular degeneration, high myopia, and trauma,
- penetrating ocular trauma,
- ruptured retinal macroaneurysm,
- Valsalva maneuver,
- blood dyscrasia such as sickle cell disease and coagulopathies,
- necrotic tumors [1],
- systemic hypertension [2].

Vessels may rupture or bleed as a result of abnormal subretinal neovascularization or arteriosclerotic degeneration in systemic hypertension causing spontaneous subretinal hemorrhage.

Currently, no data are available regarding the incidence and prevalence of spontaneous hemorrhagic retinal detachment resulting in acute angle closure glaucoma. However, there are only a few case reports about this rare ocular disorder. Spontaneous massive subretinal hemorrhage from hypertension and diabetes mellitus resulting in secondary angle closure has not been reported.

The aim of this paper is to report a case of acute angle closure glaucoma from spontaneous massive hemorrhagic retinal detachment from a systemic vascular pathology that is hypertension and diabetes mellitus.

## Case description

A 52-year-old woman came in our emergency room for consult due to sudden painful loss of vision of the right eye. The History of the present illness started 1 week prior to consult, the patient noted a sudden blurring of vision of the right eye described as curtain loss of vision. No history of trauma, pain, flashes, and floaters was noted, and neither consult done nor medication taken. Three days prior to consult, the patient noted a total loss of vision of the right eye associated with intractable pain, eye redness, headache, nausea, and vomiting, which prompted consult.

**Figure 1:**
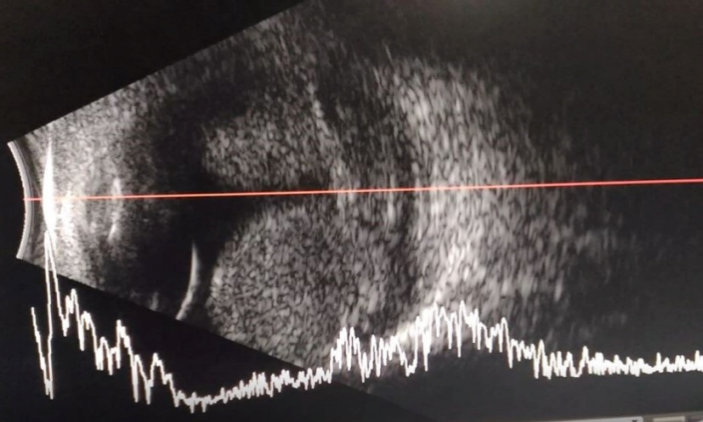
Photomicrograph of the ocular ultrasound of the right eye

The patient had no known systemic diseases except for well-controlled bronchial asthma and hypertension. The patient was maintained on losartan 50 mg twice a day with poor compliance and control. The patient came in with no light perception for the right eye and 20/20 for the left eye. The blood pressure at the time of consult was at 240/100. Gross examination and extraocular muscles were normal. The right pupil was 6 mm non-reactive to light, and the left pupil was 3 mm brisk reactive to light with positive reverse relative afferent pupillary defect. Slit lamp examination of the right eye revealed shallow chambers with moderate corneal edema with microcyst and an intraocular pressure (IOP) of 60. On gonioscopy of the right eye, the angles on all quadrants were closed. Indirect ophthalmoscopy of the right eye revealed total retinal detachment with large subretinal hemorrhage. The examination of the left eye showed a formed chamber with an IOP of 12 and a cup-to-disc ratio (CDR) of 0.4 with arteriovenous crossing changes. On gonioscopy of the left eye, the angles on all quadrants were open to ciliary body band. The right eye had poor streak on refraction, while the left eye’s refraction was –0.50 D sphere. Ocular ultrasound of the right eye (Figure 1 (Fig. 1)) revealed massive hemorrhagic retinal detachment and could not totally rule out the presence of an intraocular mass. The patient was assessed to have secondary angle closure from massive subretinal hemorrhage probably from intraocular mass metastasis of the right eye. Acetazolamide 250 mg four times a day and topical brimonidine one drop three times a day were started; however, the IOP was decreased only to 42. The blood pressure was controlled acutely with intravenous nicardipine to 170/90, then was shifted to amlodipine 100 mg once a day and losartan 100 mg twice a day. The plan was for enucleation of the painful blind eye for histologic diagnosis and for systemic work-up of the primary origin of malignancy. Since there was the clinical suspicion of a metastasis but no primary site of origin could be found, the histologic diagnosis would point to the right direction in finding a primary tumor and treating the patient for the presumed malignancy. 

Systemic and metabolic laboratory work-up revealed normal results except for elevated glycosylated hemoglobin 7.6 (3.5–6.0%) and fasting blood sugar 8.0 (4.1–5.9 mmol/L), assessing the patient with newly diagnosed type 2 diabetes mellitus. The patient was started on metformin 500 mg once a day. Mammography and transvaginal ultrasound revealed normal results. Chest radiography also showed normal results. 

**Figure 2:**
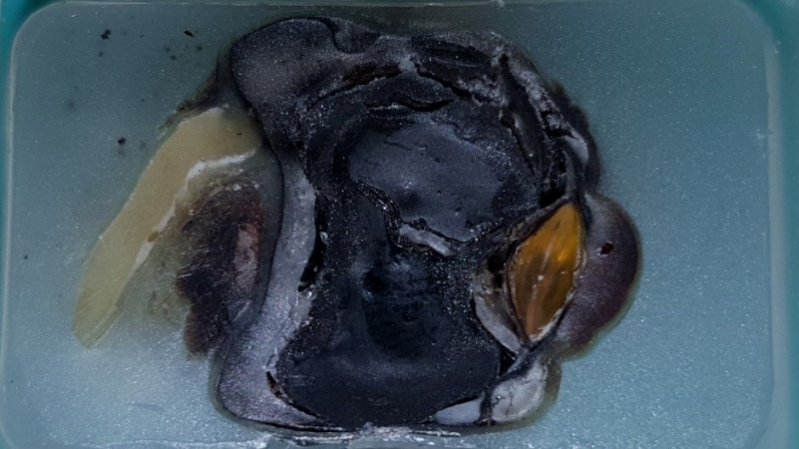
Gross antero-posterior section of the right eye embedded in paraffin block

**Figure 3:**
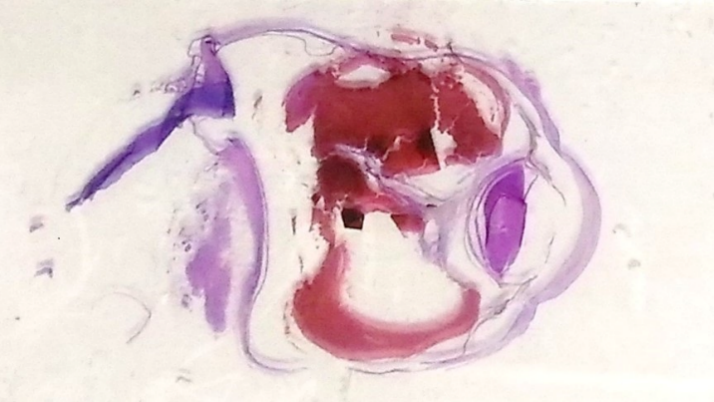
Histologic section of the right eye showing hyphema and posterior synechiae, completely closed angles, and total retinal detachment and massive subretinal hemorrhage.

Enucleation was done, and histopathology revealed subretinal hemorrhage and secondary angle closure glaucoma with no intraocular mass (Figure 2 (Fig. 2) and Figure 3 (Fig. 3)).

However, cranial and abdominal contrast enhanced computed tomography scan was done post-enucleation revealing no intracranial and abdominal mass. Hence, we were arriving at a final diagnosis of secondary acute angle closure glaucoma from spontaneous massive hemorrhagic retinal detachment secondary to hypertension and diabetes mellitus.

## Discussion

Spontaneous subretinal hemorrhage can be due to a myriad of causes, but for our patient, it was secondary to a systemic vascular pathology that is hypertension and diabetes mellitus. The mechanism of angle closure is the abrupt anterior displacement of the lens-iris diaphragm secondary to a massively detached retina and choroid [3]. The ocular pathology section of the globe as seen in Figure 2 (Fig. 2) and Figure 3 (Fig. 3) showed that the subretinal hemorrhage pushed the retina anteriorly and displacing the lens-iris diaphragm against the cornea obliterating the anterior chamber.

In this patient, a woman of middle age with no previous ocular symptoms and a normal left eye presenting with sudden hemorrhagic retinal detachment and secondary angle closure glaucoma, an intraocular mass or tumor from metastasis had to be ruled out first; hence, it was important to work up the patient for possible sources of metastasis. A small intraocular tumor could be ruled out by multiple or serial section of the eye ball.

The uveal tract is the most vascularized part of the eye; hence, hematogenously spreading carcinomatosis most often lodges in this site first [4]. The most common origins of ocular metastases are as follows: breast (47%), lung (21%), and gastrointestinal tract (4%) [4]. Affected predominantly is the choroid with an incidence of 81%; this is followed by iris (9%), optic disc (5%), and ciliary body (2%) [5].

All cases of spontaneous subretinal haemorrhage reported in the literature had at least one predisposing factor, which includes anti-coagulant therapy, blood dyscrasia or systemic hypertension [6]. Ocular risk factors predisposing to spontaneous subretinal hemorrhage include macular subretinal neovascularization or retinal vascular anomalies [7]. In our patient, preoperative examination of the other eye revealed no signs of macular degeneration. In patients with recent intraocular surgery, a massive suprachoroidal hemorrhage should be suspected. Chen et al. found out that suprachoroidal hemorrhage after intraocular surgery may not always follow a rapid course and did not always follow a hypotensive state. Retrolental hematoma gradually accumulate up to 2 to 4 weeks under normal intraocular pressure [7]. In the study of Berrocal et al., a disciform scar from age-related macular degeneration accounted for more than half of the cases of spontaneous subretinal hemorrhage. Other causes they noted were as follows: retinal macroaneurysms, presumed ocular histoplasmosis syndrome, trauma, Valsalva retinopathy, idiopathic central serous choroidopathy, diabetic retinopathy, and choroidal rupture [8]. Diabetic retinopathy mostly presents bilaterally. In 5–10% of diabetic patients, it may present unilaterally or asymmetrically [9]. Spontaneous hemorrhagic subretinal detachment from hypertension and diabetes has not been reported. A sudden high elevation of blood pressure can cause myocardial infarction in 11% of patients, cerebral infarction or haemorrhage in 7% of patients, acute kidney injury in 31% of patients, and less commonly, hypertensive encephalopathy or microangiopathic haemolytic anemia [10].

Medical therapy with ocular hypotensive agents is ineffective in these cases. Most reported cases underwent enucleation because of intractable eye pain. In other cases, retrobulbar alcohol injection or cyclodestructive laser procedures may be offered. Prolonged severe elevation of intraocular pressure causes irreversible ischemic damage to the intraocular structures; hence, visual prognosis is poor [6].

Ocular B-mode ultrasonography is an important ancillary procedure when ophthalmoscopy secondary to opacification of the lens or vitreous hemorrhage is impossible. The eye is examined through closed eyelids using a high-frequency (7.5 to 13.0 MHz) linear transducer. This tool is widely available and provides high-resolution images with real time dynamic study. In contrast, the computed tomography (CT) and magnetic resonance imaging (MRI) have poorer spatial resolution and limited role in the study of the vitreous, retina, and choroid, but are still useful in some ocular and orbital conditions. Subretinal hemorrhage presents as echogenic material in the subretinal space. In acute settings, the retina appears to be thin and mobile on dynamic scanning; but in chronic detachment, it appears as rigid triangular sign. Some entities mimic tumors on ultrasound, these includes age-related macular degeneration, subretinal hemorrhage, and macular edema due to the similar sonologic appearance [11].

In conclusion, hemorrhagic retinal detachment results from benign and malignant processes causing a sudden painful loss or decrease in vision. Breast, lung, and gastrointestinal malignancy are the most common causes of choroidal metastasis that may result in hemorrhagic retinal detachment. Thus, the importance of excluding these life-threatening conditions should be considered first. It is of utmost importance to recognize the various etiologies of this disease process to be able to do all appropriate diagnostic examination and treatment regimen, thereby maximizing the restoration of vision and quality of life of the patients. This case suggests that hypertension and diabetes mellitus may cause vessel wall changes resulting in spontaneous massive subretinal hemorrhagic retinal detachment resulting in secondary angle closure glaucoma. Therefore, ophthalmologists and physicians should be aware of this possible complication and must rule out other possible malignant etiologies. 

## Notes

### Literature search

PubMed was searched for English-language articles on March 5, 2017, using the following terms: subretinal hemorrhage, angle closure glaucoma, hypertension, diabetes mellitus, and retinal detachment. Sources in retrieved articles were cross-referenced.

### Acknowledgments

The authors thank the Ocular Oncology and Glaucoma Services for the support and guidance in making this paper.

### Competing interests

The authors declare that they have no competing interests.

### Ethical consideration

The case report is a minimal risk study which was conducted in full compliance with the principles of the 7^th^ iteration of the Declaration of Helsinki and Good Clinical Practice of the WHO. All identifying patient information was kept confidential. Informed consent from the patient was obtained prior to inclusion in the study.
